# Fever and Ulcerative Skin Lesions in a Patient Referred for Altered Mental Status: Clinical and Microbiological Diagnosis of Ulceroglandular Tularemia

**DOI:** 10.3390/tropicalmed7090220

**Published:** 2022-09-02

**Authors:** Loukas Kakoullis, Justin Pitman, Lydia Flier, Robert Colgrove

**Affiliations:** 1 Department of Internal Medicine, Mount Auburn Hospital, Cambridge, MA 02138, USA; 2 Harvard Medical School, Boston, MA 02115, USA; 3 Department of Emergency Medicine, Mount Auburn Hospital, Cambridge, MA 02138, USA; 4 Division of Infectious Diseases, Mount Auburn Hospital, Cambridge, MA 02138, USA

**Keywords:** *Francisella tularensis*, ulceroglandular tularemia, tick-borne disease, Faget sign

## Abstract

Background: Tularemia is a devastating disease that affects multiple organ systems and can have several different presentations. In its most frequent form—that of ulceroglandular tularemia—a detailed history and physical examination can enable a physician to make the diagnosis clinically, leading to the prompt initiation of the appropriate antibiotic treatment. Detailed Case Description: A 63-year-old man was brought by ambulance to the emergency department for an evaluation of an altered mental status noted by his psychiatrist at a telehealth appointment. A physical examination revealed a fever and two ulcerative lesions with a central eschar on his left leg (of which the patient was unaware) with ipsilateral tender inguinal lymphadenopathy. When asked, the patient recalled visiting Martha’s Vineyard and having removed ticks from his legs. Gentamicin was administered on the clinical suspicion of ulceroglandular tularemia. Blood and skin lesion cultures grew Gram-negative rods, which were confirmed to be *Francisella tularensis* on hospital day eight, and the patient fully recovered. Conclusion: This case highlights the importance of clinician perception of altered mental status as a key alarm sign, the necessity of a thorough physical exam independent of the chief compliant in the emergency department, and the essential role of pattern recognition by front-line providers for the appropriate management of uncommon but serious infections such as tularemia.

*“The rabbit’s dreamy eyes grow dreamier*
*As he quietly gives you tularemia.”*
A Bulletin Has Just Come In
*-Ogden Nash, 1946*

## 1. Introduction

Tularemia is an infrequent but potentially devastating disease, with substantial morbidity and mortality if untreated. The diagnosis requires a high degree of clinical suspicion. We present a case of ulceroglandular tularemia in a patient presenting with an altered mental status, initially unaware of his ulcerative skin lesions due to his confusion.

## 2. Detailed Case Description

A 63-year-old man was brought to the emergency room by ambulance after his long-term psychiatrist noted that he was disoriented and confused during a routine telehealth appointment. His past medical history was significant for seizures, right hemianopia, recurrent deep vein thrombosis, and an episode of pulmonary embolism secondary to a prothrombin gene mutation, resected thymoma, osteopenia, anxiety, and depression. In the emergency department, the patient reported difficulty sleeping for the past four days and one episode of diarrhea. He was very tangential in his responses and denied having any other symptoms. He specifically declined a recollection of fever, headaches, neck stiffness, or myalgias, and had not noticed any rashes or skin lesions.

**Figure 1:**
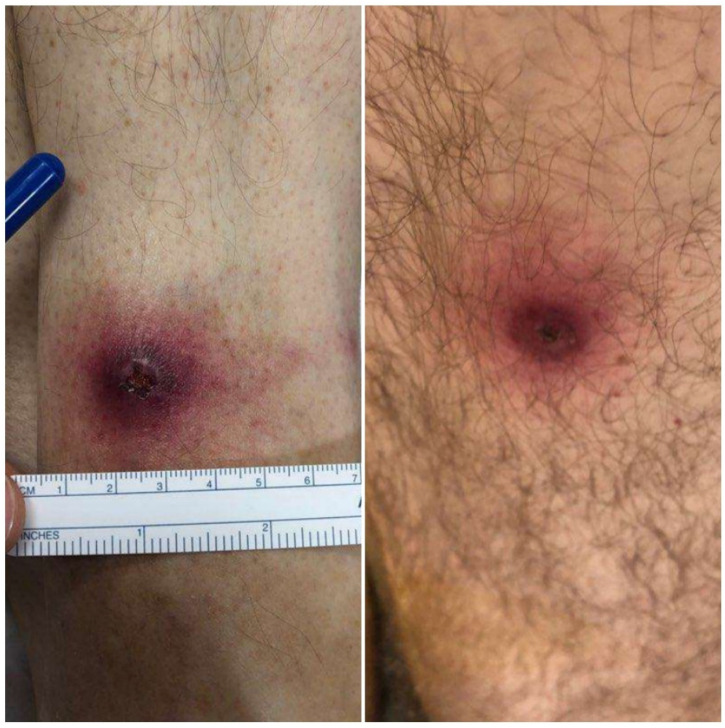
Ulcerative lesions with a central eschar on the patient’s left distal tibia (**left**) and left posterior thigh (**right**).

On arrival, his temperature was 39.3 °C, his blood pressure was 116/88, his pulse was 88, his respiratory rate was 18, and he had an oxygen saturation of 93%. His neck was supple, and a cardiopulmonary exam was normal. The physical examination was significant for two discrete ulcerative lesions with a central eschar on the left leg (Figure 1) and associated tender left inguinal lymphadenopathy. The patient was unaware of their existence, despite one being in a readily visible location slightly above his ankle, but when made aware of their location, he recalled removing ticks from both sites during a trip to Martha’s Vineyard two weeks prior.

The history of a recent tick exposure in Martha’s Vineyard, a location with a heavy burden of tick-borne infections [1,2], in combination with a fever, ulcerative lesions with a central eschar following tick exposure, and associated inguinal lymphadenopathys was suggestive of ulceroglandular tularemia. The presence of decreased oxygen saturation raised concerns for pulmonic tularemia, which has been observed in Martha’s Vineyard [3].

Infectious disease was urgently consulted in the emergency room. Using appropriate personal protective equipment due to the risk of aerosolizing bacteria, a wound culture was taken from the eschar. The lab was alerted to the possibility of *Francisella* infection, as *Francisella* represents a significant risk to laboratory staff. Easily aerosolized, with an extremely small effective inoculum size required to produce a severe clinical infection, *Francisella* must be handled with appropriate biosafety-level precautions [4,5]. The differential included a rickettsial pathogen, such as *Rickettsia parkeri*, an atypical presentation of an infection by *Bartonella henselae*, *Sporothrix schenckii*, or *Mycobacterium ulcerans*, toxin-producing stains of *Staphylococcus aureus* or *Streptococcus pyogenes*, or a toxin/venom-mediated arthropod bite wound. Viral or bacterial meningitis/encephalitis was considered. A co-infection with other tick-borne illnesses, including Lyme disease, ehrlichiosis, anaplasmosis, babesiosis, and Powassan virus encephalitis, was also considered.

An initial laboratory workup showed thrombocytopenia, kidney dysfunction, elevated inflammation markers, and a combination of elevated AST, CPK, and LDH, which was suggestive of muscle injury (Table 1).

A chest radiograph demonstrated minimal linear opacities in the left lung base. A head computed tomography showed an old infarct, but no acute pathology. Blood cultures, a blood parasite smear, a tick-borne disease molecular panel, and antibody titers for *Francisella tularensis*, *Anaplasma phagocytophilum*, *Ehrlichia chaffeensis*, *Babesia microti*, *Rickettsia rickettsia*, and *Rickettsia typhi* were drawn. Rapid antigen tests for influenza A and B, as well as a SARS-CoV-2 RNA PCR, were negative. The blood parasite smear was negative.

The patient’s mental status improved after the administration of antipyretics and defervescence, suggesting that the alteration was less likely to be secondary to the CNS involvement and more likely to be due to the fever itself. A regimen consisting of gentamycin, ceftriaxone, and doxycycline was initiated to cover for tularemia as well as other tick-borne and/or rickettsial pathogens.

The following day, the patient’s temperature had declined to 37.8 °C. His vital signs showed a pulse–temperature disassociation, with a relative bradycardia of 52 beats per minute, while his mental status had normalized and he was able to answer questions appropriately. He had a complete resolution of the fever by the third day. The blood cultures showed no growth and ceftriaxone was stopped. The bradycardia continued, with heart rates in the 40 s, increasing to the high 60 s while ambulating.

**Figure 2:**
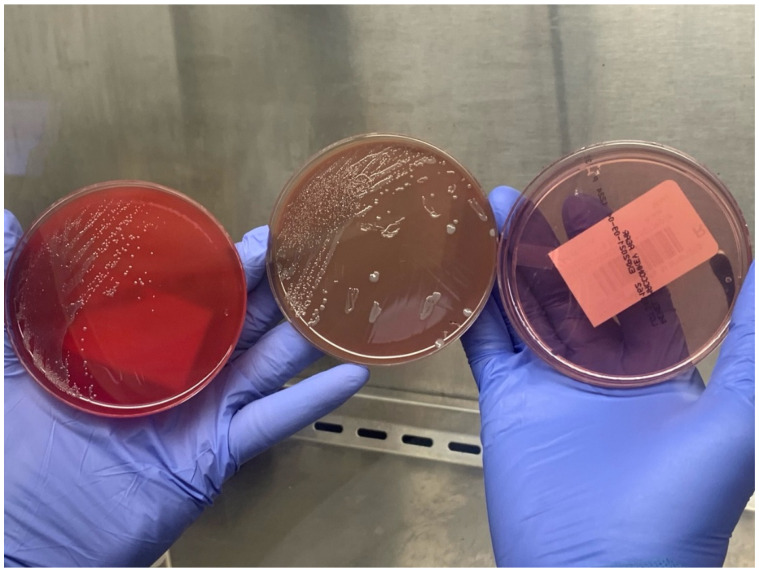
Small, slow-growing bacterial colonies, with augmented growth on chocolate agar and no growth on MacConkey agar.

**Figure 3:**
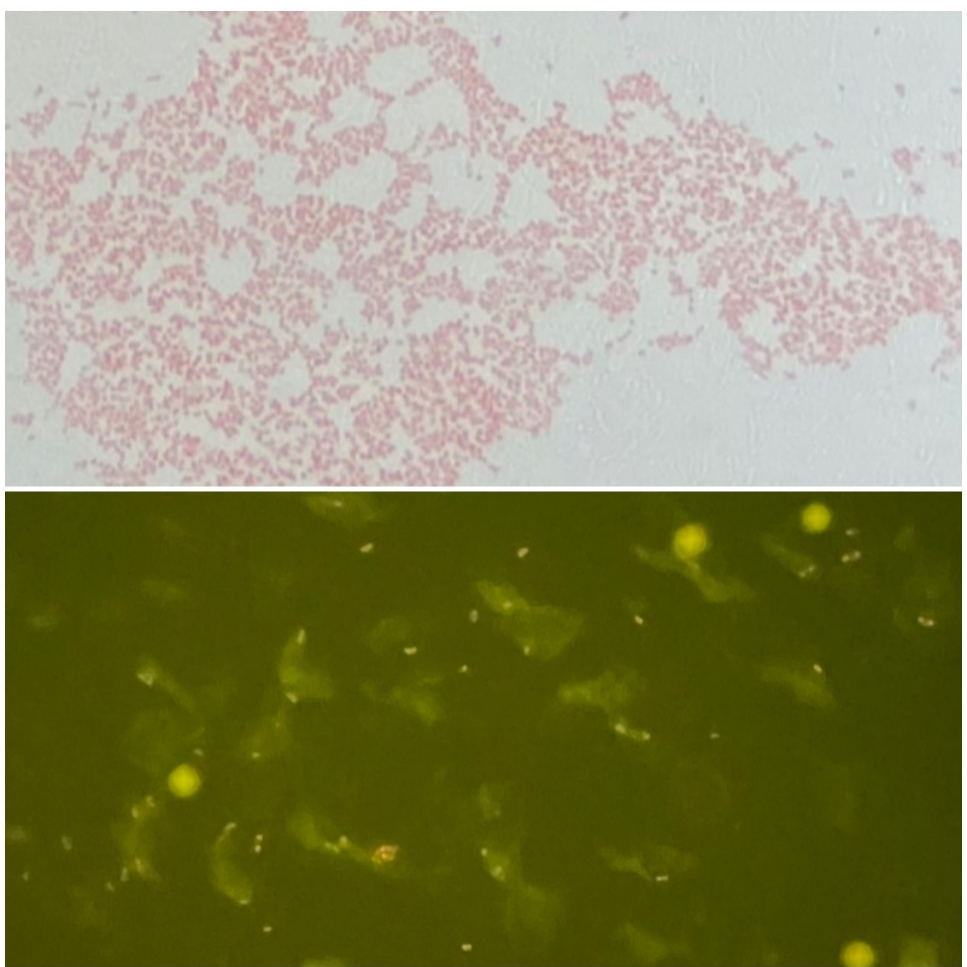
Gram stain (**top**) and acridine orange fluorescence microscopy (**bottom**) of bacteria isolated from blood cultures. Organisms appear as small, faintly-staining Gram-negative coccobacilli on a Gram stain, and as brightly fluorescent coccobacilli with the acridine orange stain.

On day four, Gram-negative coccobacilli (Figure 2) were isolated from a swab culture obtained from one of the lesions. The organisms were only very weakly visible on a Gram stain, but were more apparent when using acridine orange fluorescence microscopy (Figure 3). The tick-borne disease molecular panel (including PCR for *Ehrlichia chaffeensis*, *Babesia microti*, *Borrelia miymotoi*, *Anaplasma phagocytophilum*, and *Borrelia burgdorferi*) as well as the antibody titers for *A. phagocytophilum*, *E. chaffeensis*, *B. microti*, *R. rickettsii*, and *R. typhi* were negative; therefore, doxycycline was discontinued.

On day five, the admission blood cultures showed Gram-negative coccobacilli, while on day eight, PCR and matrix-assisted laser desorption/ionization time-of-flight mass spectrometry (MALDI-TOF) confirmed the skin lesion isolate as being *F. tularensis*.

The patient’s condition continued to improve, as did his kidney function, thromboyctopenia, and transaminases. A transthoracic echocardiogram, obtained due to the continuing bradycardia, was otherwise normal. Given the clinical improvement, gentamicin was changed to oral ciprofloxacin in case of a contribution to bradycardia. The patient was discharged to home on hospital day ten, upon completion of the antibiotics. On the day of discharge, the *F. tularensis* antibody titers obtained on the day of admission resulted in <1:20 (non-reactive). Shortly after discharge, the blood culture isolates were identified as *F. tularensis*.

On a follow-up visit 3 weeks later, the patient was doing well. His cutaneous lesions were healing and his inguinal lymphadenopathy had resolved. He was still bradycardic with a heart rate of 53, but remained asymptomatic. The transaminases and CRP had normalized. Convalescent *F. tularensis* titers obtained at that visit were 1:640 (positive result ≥ 1:160).

## 3. Discussion

The New England area is home to several tick species, which act as vectors for multiple pathogens. As such, tick-borne illnesses are a common occurrence during the summer months in this area. The vectored pathogens include *Borrelia burgdorferi*, *Anaplasma phagocytophilum*, *Babesia microti*, *Rickettsia rickettsii*, *F. tularensis*, and the Powassan virus [6,7,8]. While *F. tularensis* may be found in several locales in New England [9], Martha’s Vineyard (an island off the coast of Massachusetts) is notable as the location of an outbreak of pneumonic tularemia in 2000 [3].

This case highlights the importance of a thorough history and physical exam in the work-up of acutely ill patients, illustrated by the fact that the fever, skin lesions, and tender regional adenopathy were not among the initial presenting complaints. In addition, the recognition of the ulcerative skin lesions and regional adenopathy in a patient with tick exposure in an endemic area was essential for the prompt instigation of the appropriate antimicrobial therapy. Furthermore, pattern recognition and appropriate clinical suspicion allowed laboratory personnel to be notified and to follow the appropriate biosafety protocol.

In addition to the salient presentation, the patient’s marked thrombocytopenia was also suggestive of an unusual pathogen. Although seen in a number of infections, such as malaria, dengue, and typhus, this degree of thrombocytopenia would be unusual for most bacterial infections outside of fulminant sepsis, which did not fit the clinical picture here. The transaminitis noted in this case was another non-specific finding, but along with the low platelet count, would be consistent with tularemia.

The pathogen *F. tularensis* is divided into four subspecies, of which two are pathogenetic to humans: *F. tularensis* subsp. *tularensis* (type A), which is highly virulent, and *F. tularensis* subsp. *holarctica* (type B), which exhibits moderate virulence. *F. tularensis* subsp. *holarctica* can be found throughout the northern hemisphere, whereas *F. tularensis* subsp. *tularensis* is endemic only in North America [10,11]. Furthermore, *F. tularensis* subsp. *tularensis* has been used in the past as a biowarfare agent, and is a potential agent of bioterrorism [12].

*F. tularensis* can be transmitted via multiple routes, including direct contact with or the ingestion of infected animals, the inhalation of aerosolized bacteria, or—most commonly—from arthropods that have fed on infected animals. Several arthropods can transmit this organism, including fleas, lice, and biting flies, but in New England, the most frequent vectors are *Dermacentor* ticks. This is a distinction from the more common tick-borne infections in this region, Lyme borreliosis, anaplasmosis, and babesiosis, all of which are transmitted by *Ixodes* ticks, which can readily be distinguished from *Dermacentor* by appearance [13]. A co-infection with multiple pathogens, which is common after *Ixodes* bites, is much less likely with *Dermacentor*, the vector of *F. tularensis*. This fact, along with the positive blood cultures and negative molecular-tick-panel data, made it reasonable in this case to focus the antimicrobial therapy on *F. tularensis* with continued gentamicin alone.

An interesting manifestation in this case was the presence of a fever with relative bradycardia, or a temperature–pulse dissociation, which is known as the Faget Sign. First described the mid-19th century by New Orleans physician Jean-Charles Faget as a symptom of yellow fever [14], it has since been most classically associated with typhoid fever, but can be seen in infections by a number of other pathogens, including *F. tularensis*, *Legionella pneumophila*, and *Leptospira* species [15]. The Faget sign can suggest pathogens that may not be readily identified by culture.

Several features of the initial microbiological appearance were suggestive of *F. tularensis*. These included: (1) a long time to culture-positivity, (2) poor growth on unsupplemented media, (2) a tiny coccobacillary shape, and (4) weak uptake of the safranin counterstain in Gram staining. The fluorescent acridine orange stain, which binds to nucleic acids, can be useful for the detection of difficult-to-visualize organisms.

Although the blood cultures were eventually positive here, the cultures are most commonly negative. *Francisella* serology is also typically negative at the time of presentation. Both of these features (along with the slow growth and difficult cultivation from lesion cultures) highlight the importance of beginning directed therapy based on a clinical suspicion rather than waiting for the microbiologic diagnosis. In this case, the molecular analysis of the cultured isolate confirmed the diagnosis while the patient was still on antibiotic therapy, but more typically, when cultures and acute serologies are negative, convalescent serology 2–4 weeks later can be used for retrospective confirmation rather than for the diagnosis of acute infection. In contrast, rapid results can be obtained through molecular testing from samples obtained directly from the lesion, such as obtaining PCR directly from a swab sample obtained from the eschar site [16]; however, this modality is not available at our institution.

Although the mortality rate from tularemia in recent times has been <4% with appropriate therapy, historical data suggest that severe untreated infection can carry a much higher risk of death, exceeding 50% in some forms of the disease [17]. The referring provider’s recognition of and response to the patient’s altered mental status was critical, since the patient’s altered mental status precluded him from seeking care on his own.

## 4. Conclusions

Tularemia, given its clinical severity, complex presentation, risk to lab personnel, and possible use of the causative organism as a bioweapon, is a dangerous infection. Although relatively uncommon compared to other tick-borne illnesses in New England, clinicians practicing in endemic areas (or seeing patients who travel there) should be alert to features that are suggestive of a *Francisella* infection when evaluating acutely ill patients. Since many commonly used empiric antibiotics have little activity against this organism, the prompt consideration of tularemia and the use of appropriate antibiotics may be life-saving.

## Figures and Tables

**Table 1:** Laboratory parameters on admission.

Laboratory Parameter	Value
WBC	9430 cells/mL
Hb	14.1 g/dL
PLT	74,000 cells/mL
Na	130 mmol/L
K	3.8 mmol/L
HCO_3_	19 mmol/L
BUN	42 mg/dL
Cr	1.4 mg/dL
ALT	26 U/L
AST	75 U/L
Total bilirubin	0.9 mg/dL
INR	3.2
LDH	1384 U/L
CPK	507 U/L
CRP	449.5 mg/L
ESR	76 mm/h

## Data Availability

All data pertaining to the current work are included within this manuscript.
